# Genetic variation and population structure of *Diaphorina citri* using cytochrome oxidase I sequencing

**DOI:** 10.1371/journal.pone.0198399

**Published:** 2018-06-21

**Authors:** A. Fuentes, W. E. Braswell, R. Ruiz-Arce, A. Racelis

**Affiliations:** 1 Department of Biology, University of Texas Rio Grande Valley, Edinburg, TX, United States of America; 2 USDA APHIS CPHST Mission Lab, Edinburg, TX, United States of America; National Cheng Kung University, TAIWAN

## Abstract

Citrus greening disease, or huanglongbing (HLB), is currently one of the most devastating diseases of citrus. The bacteria thought to be responsible for the disease, *Candidatus* Liberibacter asiaticus impact the majority of commercial citrus species worldwide. These bacteria are transmitted by the Asian citrus psyllid (ACP), *Diaphorina citri* Kuwayama, which is now found in most citrus growing regions. With no known cure, ACP-vectored HLB is responsible for significant economic losses to the global citrus industry. A better understanding of the global genetic diversity of *D*. *citri* would improve current and future pest management and mitigation programs. To assess the genetic diversity of *D*. *citri* in worldwide collections, a total of 1,108 sequences belonging to ACP gathered from 27 countries in the Americas, the Caribbean, Southeast and Southwest Asia were examined for the study. 883 *D*. *citri* came from 98 locations in 18 different countries, and were sequenced using a 678bp fragment of the mitochondrial cytochrome oxidase I (COI) gene. Additionally, 225 previously-reported *D*. *citri* COI sequences, were also included in our analysis. Analyses revealed 28 haplotypes and a low genetic diversity. This is in accordance with previous reports on the little diversity of *D*. *citri* in worldwide populations. Our analyses reveal population structure with 21 haplotypes showing geographic association, increasing the resolution for the source estimation of ACP. This study reveals the distribution of haplotypes observed in different geographic regions and likely geographic sources for *D*. *citri* introductions.

## Introduction

The Asian citrus psyllid (ACP), *Diaphorina citri* Kuwayama [Hemiptera: Liviidae], is a phloem-feeding insect that in addition to causing physical damages to the plant, is the most effective vector of *Candidatus* Liberibacter asiaticus, and *Candidatus* Liberibacter americanus, the causal agents responsible for Huanglongbing (HLB) disease [1]. Nymphs and adults of *D*. *citri* acquire the bacterial pathogen when feeding on infected plants. Once the bacteria are inside the vector, they replicate, invade the salivary glands, and thus enable Ca. Liberibacter to infect other citrus plants through psyllid feeding [2–4]. Citrus greening disease is considered the most serious disease of citrus in the world [5], as most commercial citrus species are adversely affected by this bacterium [6]. Affected trees develop a variety of symptoms including mottling of the leaves and chlorosis, root death [7, 8], and small, discolored fruit with a bitter taste [9]. At present, the primary methods to control HLB is destructive removal of infected trees, psyllid control and plantation of Ca. Liberibacter free trees [10, 11]. Therefore, as a key vector for Ca. Liberibacter, *D*. *citri* represents a dangerous threat to regions that are still free of this disease [12].

*D*. *citri* was first described in Taiwan in 1907, and it is thought to be native to Southwestern Asia [9, 13]. Specifically, it has been suggested that *D*. *citri* evolved in India, in association with a species of orange jessamine *Murraya* [14, 15]. It has since been documented in throughout north Asia, Southwestern Asia, and southeastern Asia, and in some parts of the Middle East, causing significant damages to the citrus growing regions in these countries [16, 17]. *D*. *citri* was first reported in South America in 1942, and now is common to citrus growing regions in Brazil, Argentina, Venezuela, Paraguay, and Uruguay [18]. It is also pervasive in Central America, reported in Mexico, Costa Rica, Belize and Honduras [17, 19], and most of the Caribbean islands including Bahamas, Cayman Islands, Cuba, Jamaica, Dominican Republic, Guadeloupe, Abaco Island, Grand Bahama Island, U.S. A. Virgin Islands, and Puerto Rico [17, 20].

ACP has also invaded the Pacific Islands of Hawaii, American Samoa, Northern Mariana Islands, and Guam and is present in the following states of the mainland United States: Alabama, Arizona, California, Florida, Georgia, Louisiana, Mississippi, South Carolina and Texas [16, 20, 21,22]. *D*. *citri* is being actively managed across the commercial citrus growing regions of the U. S. A., including Arizona, California, Florida, and Texas, as well as non-commercial regions. In Florida alone, citrus losses associated with *D*. *citri* and citrus greening has been estimated to exceed U. S. A. $3.6 billion in a 5-year period [23, 24], and its spread continues to threaten the entire citrus industry worldwide. Minimizing further infestations by *D*. *citri* is paramount to help reduce the spread of citrus greening disease.

Knowledge of the genetic variation and population structure of an invasive pest species, such as *D*. *citri*, can help on the identification of the geographical origin of the invader, and can potentially provide insights of invasion routes. This information can also be useful for biological control programs and other pest management programs [25]. Mitochondrial DNA analysis has been widely used for phylogenetic inferences and population genetic inferences [26, 27, 28]. Previously, the global genetic diversity of *D*. *citri* from 15 different countries was studied using mitochondrial cytochrome oxidase I (mtCOI) sequencing by Boykin et al. [27]. Their study detected 8 haplotypes divided in two major haplotype groups South West Asia (SWA), and South-East Asia (SEA). They proposed that two independent introductions occurred in the New World [27], as suggested by a previous study on the genetic diversity of *D*. *citri* [22]. However, due to the limited sampling in the Old World they could not resolve the geographic origin of *D*. *citri*. The authors suggested that further sampling, especially from the Middle East and Asian countries was needed to better define the genetic diversity of this insect.

Because of the limited knowledge on the genetic diversity of *D*. *citri*, identifying the source or pathway of the Asian citrus psyllid is difficult. Additional data from geographic regions where this pest occurs is needed. The objective of this study is to: (I) examine the genetic variation of *D*. *citri* using COI sequences in samples from different geographic locations and (II) to examine these collections for population structure in order to further populate the dataset used for the pathway analysis of this pest. This information would aid in decision-making concerning pest management programs.

## Materials and methods

### Insect collection

A total of 883 *D*. *citri* samples were collected from different citrus host-plants, and made available to us by collectors from 18 different countries between 2008 and 2012 (Table 1). Additionally, 225 *D*. *citri* COI sequences deposited in GenBank by Boykin et al. [27], Lashkari et al. [29], and Chaitanya et al. [30] were included (Table 2), for resulting in a total of 1,108 individuals included in this global phylogenetic analysis.

**Table 1:** Geographical origin of *D*. *citri* samples analyzed in this study.

Site no.	Country (# of individuals)	Site	n	Latitude	Longitude	Year	Host
1	American Samoa (22)	American Samoa, Amanave Village	1	-14.32664 S	170.82886 W	2011	*C*. *limon*
2		American Samoa, Amouli Village	1	-14.27298 S	170.58499 W	2012	*C*. *aurantium*
3		American Samoa, Aoa Village	1	-14.26147 S	170.58553 W	2011	*C*. *aurantifolia*
4		American Samoa, Asili Village	1	-14.33071 S	170.79608 W	2011	*C*. *aurantium*
5		American Samoa, Auasi Village	1	-14.27056 S	170.57408 W	2012	*C*. *aurantifolia*
6		American Samoa, Fagaalu Village	1	-14.28962 S	170.68767 W	2011	*C*. *aurantifolia*
7		American Samoa, Fagaitua Village	1	-14.26783 S	170.6153 W	2011	*C*. *aurantifolia*
8		American Samoa, Fagatogo Village	1	-14.27962 S	170.69231 W	2011	*C*. *aurantium*
9		American Samoa, Faleniu Village	1	-14.32985 S	170.74478 W	2011	*C*. *limon*
10		American Samoa, Fatumafuti Village	1	-14.29742 S	170.67842 W	2012	*C*. *aurantifolia*
11		American Samoa, Futiga Village	1	-14.34895 S	170.75926 W	2011	*C*. *aurantifolia*
12		American Samoa, Leone Village	1	-14.33727 S	170.78508 W	2012	*C*. *limon*
13		American Samoa, Malaeimi Village	1	-14.32152 S	170.74036 W	2012	*C*. *aurantifolia*
14		American Samoa, Matu'u Village	1	-14.29853 S	170.68532 W	2012	*C*. *paradisi*
15		American Samoa, Nua Village	1	-14.32516 S	170.80875 W	2011	*C*. *aurantifolia*
16		American Samoa, Nuuuli Village	1	-14.31373 S	170.71385 W	2011	*C*. *reticulata*
17		American Samoa, Pago Pago Village	1	-14.27842 S	170.70582 W	2011	*C*. *aurantifolia*
18		American Samoa, Pavaia'i Village	1	-14.33314 S	170.75023 W	2011	*C*. *aurantifolia*
19		American Samoa, Tafuna Village	1	-14.33472 S	170.72968 W	2011	*C*. *limon*
20		American Samoa, Tula Village	1	-14.25253 S	170.56743 W	2011	*C*. *aurantifolia*
21		American Samoa, Utumea Village	1	-14.32851 S	170.81506 W	2012	*C*. *aurantifolia*
22		American Samoa, Utusi'a Village	1	-14.2706 S	170.61887 W	2012	*C*. *aurantifolia*
23	Argentina (15)	Argentina:Bella Vista, Corrientes	4	-28.507731 S	-59.044857 W		* *
24		Argentina:Federacion, Entre Rios	3	-30.985036 S	-57.919842 W		* *
25		Argentina:Fraile Pintado, Jujuy	4	-23.944207 S	-64.803149 W		* *
26		Argentina:Yuchan, Salta	4	-23.433330 S	-64.16667 W		* *
27	Barbados (72)	Barbados, Golden Grove	33	13.156757 N	-59450503 W	2010	*Citrus sp*.
28		Barbados, St. Michael, Clermont	39	13.145170 N	-59.617539 W	2010	*Citrus sp*.
29	Belize (9)	Belize, Stann Creek	9	16.811663 N	-88.401604 W	2009	* *
30	China (55)	China Yunnan, Xishuangbanna	4	21.11440 N	101.41234 E	2010	*M*. *paniculata*
31		China: Hong Kong, Pak Kong	3	22.379476 N	114.258182 E	2010	*M*. *paniculata*
32		China: Jiangxi Province, Chongyl	1	25.40433 N	114.18285 E	2011	*Citrus sp*.
33		China: Gangzhou	40	23.129110 N	113.264385 E		* *
34		China: Zhejaing	7	30.267443 N	120.152792 E		* *
35	Colombia (12)	Colombia: Armero Guayabal	4	5.030556 N	-74.885556 W		*C*. *aurantifolia*
36		Colombia: Municipio de Coello	4	4.354029 N	-74.863576 W		*C*. *aurantifolia*
37		Colombia: Municipio de Guamo	4	4.195320 N	-75.00753 W		*C*. *aurantifolia*
38	Costa Rica (10)	Costa Rica	10	9.748917 N	83.753428 W		* *
39	Mexico (30)	Mexico: Tijuana, Baja California	1	32.454655 N	-116.916855 W	2010	* C*.*sinensis*
40		Mexico: Tijuana, Baja California	1	32.521307 N	-116935036 W	2010	* C*. *sinensis*
41		Mexico: Hermosillo, Sonora	6	29.072967 N	-110.955919 W	2008	* *
42		Mexico: Jalpan, Queretaro	2	21.207300 N	-99.472267 W	2012	* *
43		Mexico: Nuevo Mugica, Michoacan	2	19.429241 N	-102.084917 W	2012	*C*. *aurantifolia*
44		Mexico: Puerto Vallarta, Jalisco	2	20.653407 N	-105.225332 W	2012	*C*. *latifolia*
45		Mexico: Rio Grande, Oaxaca	1	16.010481 N	-97.433525 W	2012	*C*. *latifolia*
46		Mexico: Los Mochis, Sinaloa	2	25.790466 N	-108.985882 W	2012	*C*. *aurantifolia*
47		Mexico: Ciudad Obregon, Sonora	2	27.482773 N	-109.930367 W	2012	*C*. *sinensis*
48		Mexico: Huimanguillo, Tabasco	2	17.830278 N	-93.391389 W	2012	*C*. *sinensis*
49		Mexico:Tamuin, San Luis Potosi	2	22.00000 N	-98.783333 W	2012	*C*. *sinensis*
50		Mexico: Tecoman, Colima	1	18.908889 N	-103.874722 W	2012	*C*. *aurantifolia*
51		Mexico: Tepic, Nayarit	2	21.504165 N	-104.894589 W	2012	*C*. *latifolia*
52		Mexico: Ucum, Quintana Roo	2	18.502535 N	-88.518226 W	2012	*C*. *latifolia*
53		Mexico: Cazones, Veracruz	1	20.723230 N	-97.31353 W	2010	*C*. *paradisi*
54		Mexcio: Mtz de la Torre, Veracruz	1	20.061513 N	-97.054526 W	2012	*C*. *latifolia*
55	Pakistan (374)	Pakistan: Bhalwal	39	32.275141 N	72.904714 E	2010	*Citrus sp*.
56		Pakistan: Faisalabad	13	31.418714 N	73.079107 E	2009	*Citrus sp*.
57		Pakistan: Lalian	40	31.825260 N	72.80274 E	2009	*Citrus sp*.
58		Pakistan: Mandi Bahauddin	41	32.588169 N	73.497343 E	2010	*Citrus sp*.
59		Pakistan: Sahiwal	42	30.661181 N	73.108576 E	2009	* *
60		Pakistan: SGD	41	32.003973 N	72.723141 E	2010	* *
61		Pakistan: Shahpur	18	32.286612 N	72.430253 E	2010	*Citrus sp*.
62		Pakistan: Shamasabad Jhang	39	31.592714 N	73.050454 E	2010	*Citrus sp*.
63		Pakistan: Toba Tek Singh	40	30.966667 N	72.483333 E	2010	*Citrus sp*.
64		Pakistan: Toba Tek Singh	40	30.966667 N	72.483333 E	2010	*Citrus sp*.
65		Pakistan: Multan	10	30.198381 N	71.468703 E		* *
66		Pakistan: Singh & Sargoda	11	32.113041 N	73.935097 E		* *
67	Paraguay (7)	Paraguay: Itapua	7	-26.792362 S	-55.668964 W		* *
68	Puerto Rico (59)	Puerto Rico: Isabela, Guerrero	19	18.473889 N	-67.048056 W	2010	*C*. *sinensis*
69		Puerto Rico: Arecibo	38	18.454893 N	-66.758134 W		*Citrus sp*.
70		Puerto Rico: Carolina, Vista Mar	2	18.436944 N	-65.981944 W		* *
71		Puerto Rico	2				* *
72	Reunion (4)	Reunion Island: Saint Pierre	4	-21.332838 N	55.471843 E	2011	*Murraya*
73	Saudi Arabia (4)	Saudi Arabia	4	23.885942 N	45.079162 E		* *
74	Singapore (3)	Singapore: Chinese Gardens	1	1.20358 N	103.43867 E	2010	*M*. *paniculata*
75		Singapore: Kim Seng Road Park	1	1.17604 N	103.4995 E	2010	*M*. *paniculata*
76		Singapore: Winsland Place	1	1.17952 N	103.50393 E	2010	*M*. *paniculata*
77	Thailand (29)	Thailand: NST, Twin Lotus Hotel	13	8.391284 N	99.978176 E	2010	*M*. *paniculata*
78		Thailand: Bangkok	16	13.786902 N	100.512641 E	2010	*M*. *paniculata*
79	Trinidad (19)	Trinidad: Grand Bazaar	19	37.169463 N	-104.500541 W	2010	*C*. *limettoides*
80	Uruguay (6)	Uruguay: Itapebi, Salto	6	-31.287202 S	-57.7.5548 W		* *
81	USA (153)	USA: AZ Yuma	2	32.692651 N	-114.627692 W	2010	*C*. *lemon*
82		USA: AZ Yuma	1	32.490834 N	-114.76772 W	2010	*C*. *lemon*
83		USA: LA, Kenner	1	29.996357 N	-90238538 W	2011	*Citrus sp*.
84		USA: LA, Grammercy	1	30.061528 N	-90.696012 W	2011	*C*. *unshu wi*
85		USA: SC, Port Royal	1	32.376735 N	-80.69475 W	2012	*C*. *sinensis*
86		USA: CA, Los Angeles County	3	34.052227 N	-118.243660 W	2009	* *
87		USA: CA, Imperial County	9	33.011369 N	-115.473355 W	2010	* *
88		USA: HI, Hilo, Homelani	16	19.718755 N	-155.089629 W	2010	*M*. *paniculata*
89		USA, TX, Edinburg,	81	26.301737 N	-98.163343 W	2010	*M*. *paniculata*
90		USA: TX, North East of Hidalgo	19	26.313640 N	-97.8634.16 W	2010	*Citrus sp*.
91		USA: TX, Corpus Christi	2	27.800583 N	-97.396381 W	2008	*Citrus sp*.
92		USA: TX, Presidio, Presidio County	2	29.560738 N	-104372146 W	2008	*Citrus so*.
93		USA: TX, Houston, Harris County	2	29.760427 N	-95.369803 W	2008	*Citrus sp*.
94		USA: TX, Uvalde	2	29.209684 N	-99.786168 W		* *
95		USA: TX, Tilden	2	28.461508 N	-98.549378 W		* *
96		USA: TX, San Antonio	2	29.457978 N	-98.460466 W		* *
97		USA: TX, Refugio	2	28.292619 N	-97271013 W		* *

**Table 2:** Accession numbers of *D*. *citri* sequences recovered from GenBank included in the analyses.

Country	Accession No.	Author	Country	Accession No.	Author
US: Florida	FJ190167-FJ190176	Boykin et al. 2012	Mexico	FJ190300-FJ190309	Boykin et al. 2012
	FJ190182-FJ190191		Mauritius	FJ190312-J190316	
	FJ190193-FJ190227		Reunion	FJ190317-FJ190318	
	FJ190232-FJ190259		Brazil	FJ190321-FJ190327	
	FJ190277-FJ190278		Brazil	FJ190329-FJ190333	
	FJ190310-FJ190311		Indonesia	FJ190336	
	FJ190372-FJ190377		Saudi Arabia	FJ190337-FJ190340	
US: Texas	FJ190177-FJ190181		India	FJ190342-FJ190345	
Brazil	FJ190228-FJ190231		Guadeloupe	FJ190346-FJ190356	
Puerto Rico	FJ190260-FJ190263		China	FJ190357-FJ190364	
Indonesia	FJ190263-FJ190271		China	FJ190366-FJ190369	
Indonesia	FJ190280-FJ190282		Vietnam	FJ190272-FJ190276	
Taiwan	FJ190284-FJ190287		Vietnam	FJ190278-FJ190282	
Pakistan	FJ190288-FJ190292		Pakistan	KC509561-KC509572	Lashkari et al. 2013
Thailand	FJ190293-FJ190294		India	KF702297-KF702306	Chaitanya et al. 2016
China	FJ190297-FJ190-299				

### Genomic DNA isolation, Polymerase Chain Reaction (PCR) amplification, and sequencing

Total genomic DNA was isolated from each individual using the DNeasy Blood and Tissue Kit (Qiagen, Valencia, CA). The primers (forward: DCITRI COI-L 5’- AGG AGG TGG AGA CCC AAT CT-3’) and (reverse: DCITRI COI-R 5’- TCA ATT GGG GGA GAG TTT TG-3’) designed by Boykin et al. [27] were used to amplify a partial fragment of COI from *D*. *citri*.

The Polymerase Chain Reaction (PCR) contained 16.77μl of DNAase free water, 2.50μl of 10X Taq Buffer (TaKaRa Bio Inc, Mountain View, CA), 2.60μl of dNTP mixture 2.5μM each (TaKaRa), 0.13μl TaKaRa Ex Taq polymerase (5U/μl), 1μl of each primer at 10μM and 1μl of DNA template for a total volume of 25μl. The amplification was conducted in Applied Biosystems (Foster City, CA) Gene Amp® PCR System 9700 thermal cycler and the following cycling parameters were used: 94°C for 2 minutes, followed by 35 cycles of 30 seconds at 94°C, 30 seconds at 53°C, of 1 min at 72°C extension and a final extension of 72°C for 10 minute, as described in Boykin et al. [27].

The PCR products were stained with Blue/Orange 6X loading Dye (Promega, Madison, WI) and visualized with ethidium bromide on 1.5% electrophoresis agarose gels at 90V for 90 minutes in 1X TAE buffer. Documentation of these gels was performed using UVP Gel-Doc-it^TS2^ imager (Upland, CA). Before sequencing, the PCR products were purified with Exo-SAP-IT^TM^ (Affimetrix, Santa Clara, CA), using the company protocol. Sequencing of PCR products was performed using bidirectional sequencing and 3’ BigDye-labeled dideoxynucleotide triphosphates (v. 3.1 dye terminators, Applied Biosystems, Foster City, CA, USA), and run on an ABI 3730XL DNA Analyzer with the ABI Data Collection Program v. 2.0 at the Huck Institute’s Nucleic Acid facility at Pennsylvania State University. Sequences were edited using Sequencher® v. 5.0 (Gene Coders Corp. Ann Arbor, MI). The alignment was constructed in MEGA v. 6.0 [31], using Clustal W [32]. Sequences were trimmed of primers to 678bp.

### Genetic diversity and population structure

Haplotypes (h) were identified using DnaSP v. 5.10.01 [33]. DNA Pairwise genetic distances were estimated in MEGA v.6. The number of haplotypes (h), haplotype diversity (Hd), nucleotide diversity (μ) the average number of pairwise differences (k) [34], and the Tajima’s D [35] and Fu and Li’s D and F [36] tests were determined with DnaSP v. 5.10.01 [33]. Tajima’s and Fu and Li’s tests were performed to test for neutral evolution and evaluate the potential for recent population expansion or contraction. General population genetics statistics were calculated to determine probable center of origins based on diversity. A haplotype network, which include haplotype frequencies, was constructed in PopArt [37] using the TCS statistical algorithm [38]. An analysis of molecular variance (AMOVA) was performed to determine the percentage of variation among geographic groups, among populations, and within groups, were performed in ARLEQUIN v. 3.5 [39].

### Phylogenetic and haplotypes analyses

JModelTest 2 [40] was used to identify the best substitution model for the DNA sequence data. A Bayesian phylogenetic reconstruction was then conducted using Mr.Bayes v.3.2.3 [41] in Cipres [42] allowing transitions and transversions to have different rate. The analysis was performed using a Monte Carlo Markov Chain (MCMC) method. Four chains were run independently for 10,000,000 generations with sampling conducted every 1,000 generations. The COI sequence of the hackberry petiole gall psyllid (*Pachypsylla venusta*) was selected as an out-group for the analyses (accession number: NC_006157). Additionally, a phylogenetic analysis using maximum likelihood methods was performed in PhyML v. 3.0 [43], using the nearest-neighbor interchange tree rearrangement and 1,000 bootstrap replications. Output trees were visualized in Cipres with midpoint rooting.

## Results

### Genetic variation

The final DNA sequence alignment of mitochondrial COI from 1,108 individuals was 678bp and contained a total of 29 polymorphic sites, 12 parsimoniously informative sites, and no indels. There were 18 synonymous changes and 11 non-synonymous. Unique sequences were submitted to GenBank (accession numbers: MH001373-MH001384). This resulted in 28 haplotypes shown by samples gathered from among the 26 locations analyzed for the study (Table 3). Of these haplotypes, 16/28 (57.14%) were identified from single individuals (singletons). The genetic diversity for the entire sequence dataset was low (<1%), and the maximum number of substitutions was 5bp for the most diverse haplotype (Dcit-20-Texas). Nucleotide and haplotype diversity were 0.00293 and 0.734 respectively. Four haplotypes were observed among 95.84% of these individuals. These included Dcit-1 368/1108 (33.21%), Dcit-2 158/1108(14.27%), Dcit-3 160/1108 (14.45%) and Dcit-06 376/1108 (33.96%). The average number of haplotypes per collection was 2.19 and ranged from 1 to 10 haplotypes per site for the entire dataset. The majority (92.3%) of these haplotypes were private to a single geographic region: fourteen haplotypes were unique to South West Asia (SWA), five for North and Central America (NCA), four for South East Asia (SEA), and two for the Caribbean (CAR).

**Table 3:** Geographic distribution and frequency of the 28 haplotypes of *D*. *citri* observed in this study.

Haplotype	Cluster/Group	Origin of population (n = # of individuals)
Dcit-01	B1	USA (229), Mexico (33), Belize (9), Costa Rica (10), American Samoa (22), Brazil (4), Puerto Rico (14), Saudi Arabia (8), Iran (10), Pakistan (9), India (4), China (11), Indonesia (4), Reunion (1)
Dcit-02	B2	Brazil (12), Paraguay (7), Argentina (15), Uruguay (6), China (59), Taiwan (4), Indonesia (9), Thailand (22), Singapore (3), Mauritius (5), Reunion (6) Vietnam (10)
Dcit-03	B3	USA (13), Puerto Rico (48), Guadeloupe (8), Barbados (72), Trinidad (19)
Dcit-04	B1	Mexico (6)
Dcit-05	B1	China (3)
Dcit-06	A	Colombia (12), Pakistan (361), India (3)
Dcit-07	B1	USA (1)
Dcit-08	B2	China (1)
Dcit-09	A	Pakistan (1)
Dcit-10	A	Pakistan (1)
Dcit-11	A	Pakistan (3)
Dcit-12	A	Pakistan (1)
Dcit-13	B3	Guadeloupe (3)
Dcit-14	A	Pakistan (1)
Dcit-15	A	Pakistan (2)
Dcit-16	A	Pakistan (1)
Dcit-17	A	Pakistan (1)
Dcit-18	B2	Thailand (9)
Dcit-19	B2	USA (2)
Dcit-20	B3	USA (1)
Dcit-21	B1	Mexico (1)
Dcit-22	B2	China (1)
Dcit-23	B3	India (2)
Dcit-24	A	India (1)
Dcit-25	A	India (1)
Dcit-26	A	India (1)
Dcit-27	A	India (1)
Dcit-28	A	India (1)

### Phylogenetic analyses

**Fig 1:**
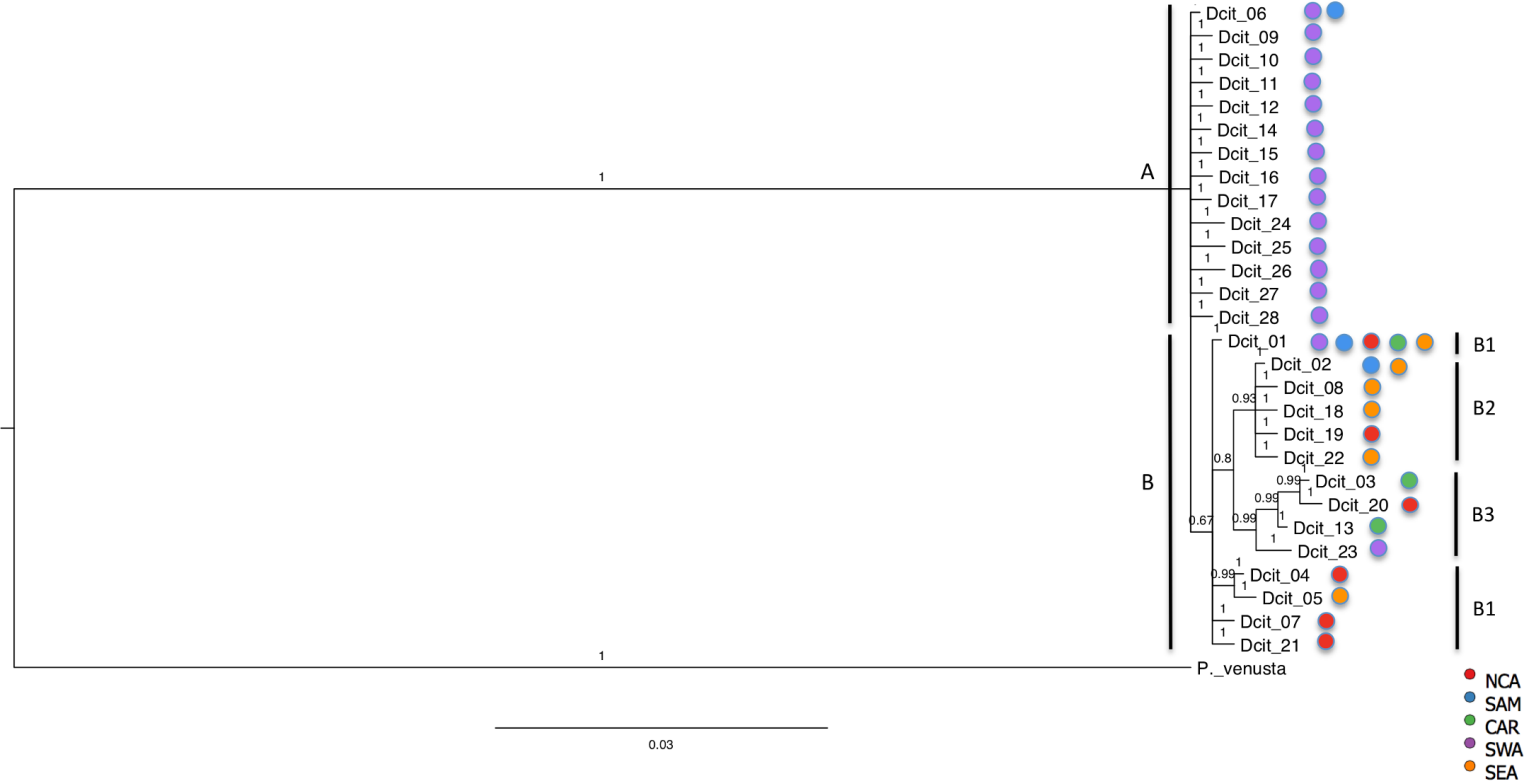
Bayesian tree showing the phylogeny of the 28 haplotypes of *D*. *citri*. Two major clusters are observed: A and B, where B is further divided in groups: B1, B2 and B3.

**Fig 2:**
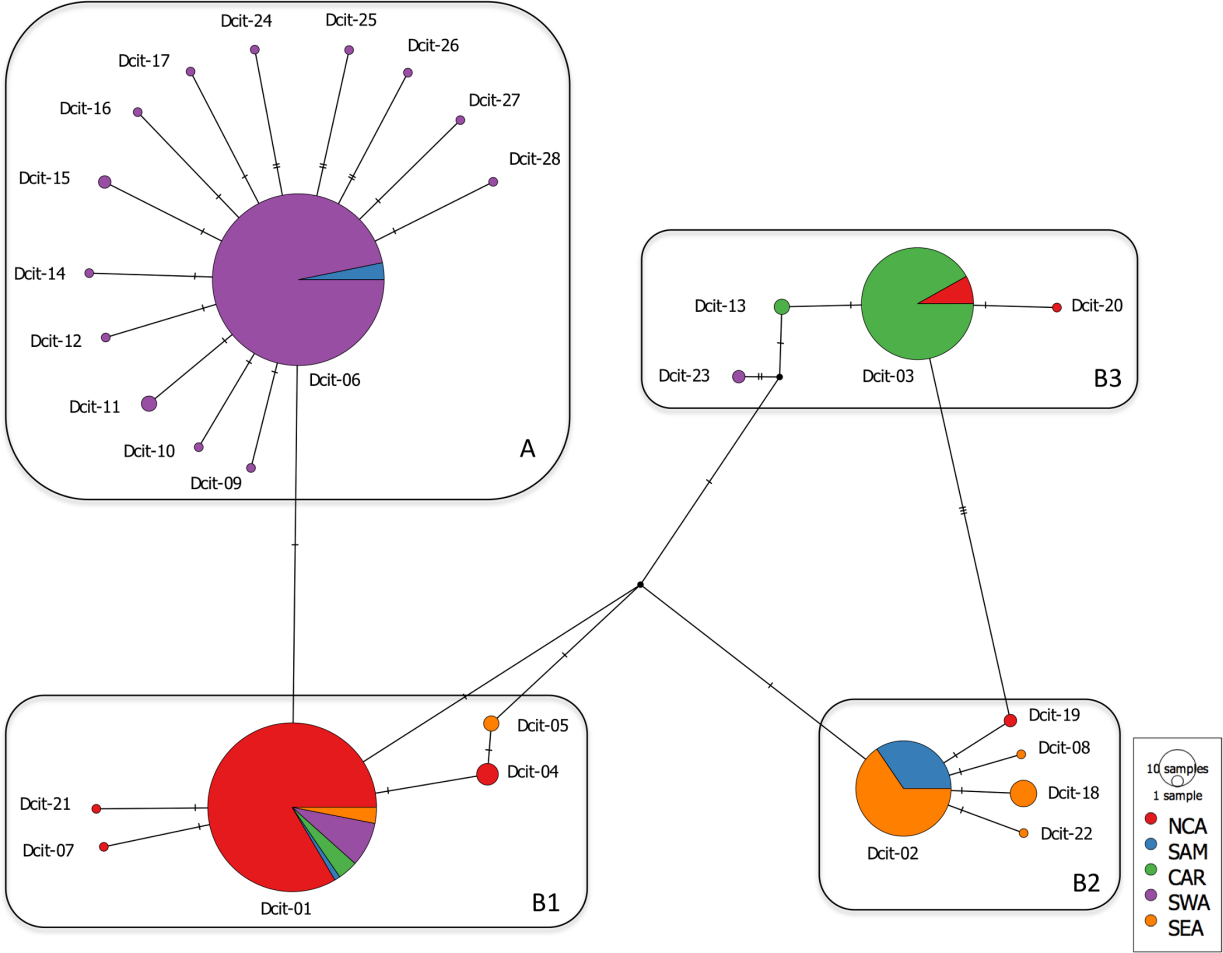
Haplotype TCS network showing the genetic relationship of 28 haplotypes recovered from this study. Circles represent haplotypes and connecting lines represent patterns of relationship. Hash marks represent additional mutational steps. Colored circles represent haplotypes observed in the regions associated with their color (NCA–North and Central America, SAM–South America, CAR–The Caribbean, SWA–South West Asia, and SEA–South East Asia). Size of each colored circle is proportional to the observed frequency of the haplotype, and proportion of samples from each region is indicated by the proportion of color associated with that region. Black circles represent inferred haplotypes.

The HKY85 + I + G substitution model was selected as the best model based on Akaike Information Criterion ranking. Modeltest revealed a proportion of invariable sites of 0.1860, a transition/transversion ratio of 1.6314, and gamma shape parameter of 0.6100. The Bayesian (Fig 1) and ML (not shown) phylogenetic analyses showed concordant topologies. The Bayesian analysis and the constructed phylogenetic network (Fig 2) support the presence of geographic structure for *D*. *citri*. This analysis revealed strong support for genetic clusters and groups. In the Bayesian analysis, two major genetic clusters were observed (A and B). Cluster A contains 14 haplotypes and shows strong support, and cluster B contains 14 haplotypes with moderate support (.67). Three genetic groups (B1, B2, and B3) were observed in cluster B. Each of these three groups showed strong branch support (Fig 1).

### Population structure

Geographic association was observed for genetic clusters and groups structure (Fig 1). For example, in Pakistan 372/381 (97.64%) fall within cluster A, as well as 10/14 (71.43%), of the individuals from India, and 13/13 (100%) of the individuals from Colombia. This cluster included fourteen haplotypes: Dcit-06, Dcit-09 through Dcit12, Dcit-14 through Dcit-17, and Dcit-24 through Dcit-28. The haplotype network shows a star-like shape arrangement seen among haplotypes from cluster A. Dcit-06 is the most common central haplotype for this cluster with the remaining closely related haplotypes present in by no more than three individuals. The haplotypes are not more than three mutational steps from the central haplotype (Dcit-06).

Cluster B consisted of groups B1, B2 and B3. Group B1, includes five haplotypes: Dcit-01, Dcit-04, Dcit-05, Dcit-07 and Dcit-21. Dcit-01 is the most geographically distributed haplotype seen in the study. The remaining four haplotypes are not more than four mutational steps from the most frequent haplotype (Dcit-01), they are locally restricted to a single collection site each. This group included individuals from 14 of the 26 countries; U. S. A. 230/246 (93.49%), Mexico 40/40 (100.0%), Belize 9/9 (100%), Costa Rica 10/10 (100%), American Samoa 22/22 (100.0%), Saudi Arabia 8/8 (100.0%), Iran 10/10 (100.0%), Brazil 4/16 (25.00%), Puerto Rico 14/62 (22.58%), Pakistan 9/381 (2.36%), India 4/14 (28.57%), China 11/75 (14.67%), Indonesia 4/13 (30.77%), Reunion 1/7 (14.29%).

Group B2 includes five haplotypes (Dcit-02, Dcit-08, Dcit-18, Dcit-19, and Dcit-22). The most common and widespread haplotype is Dcit-02. The other four haplotypes are no more than two mutational steps away from Dcit-02, and are limited to a single collection site each. This group contain 7/7 (100.0%) of the individuals from Paraguay, 15/15 (100.0%) of the samples from Argentina, 6/6 (100.0%) of the samples from Uruguay, 61/75 (81.33% of the individuals from China, 4/4 (100.0%) of the specimens from Taiwan, 2/246 (0.81%) of the samples collected in the US, 10/10 (100.0%) of the samples from Vietnam, 9/13 (69.23%) of the specimens from Indonesia, 31/31 (100.0%) of the samples from Thailand, 3/3 (100.0%) of the individuals from Singapore, 5/5 (100.0%) of the individuals collected in Mauritius, and 6/7 (85.71%) of the specimens from Reunion.

Group B3 included haplotypes Dcit-03, Dcit-13, Dcit-20 and Dcit-23. The most common haplotype is Dcit-03. The three other haplotypes were not more than six mutational steps away and were limited to a single collection site each. This group contains 13/246 (5.28%) of the individuals from the US, 48/62 (77.42%), of the individuals from Puerto Rico, 8/11 (78.73%) of the samples collected in Guadeloupe, 72/72 (100.0%), of the individuals gathered in Barbados, and 19/19 (100.0%), of the individuals from Trinidad.

Using natural geographic barriers, previously posed hypotheses, and observed patterns of distribution, the data were delineated into five populations: North and Central America (NCA), South America (SAM), The Caribbean (CAR), South West Asia (SWA) and South East Asia (SEA). The AMOVA confirmed the significance of population structure for the five geographic populations (Table 4). The AMOVA rejected the null hypothesis that the five populations are homogeneous. The highest source of variation (75.92%) was observed within regions, according to the five populations we mention above, for those combinations we tested. Variation was also observed among populations (10.05%), and within populations (14.03%).

**Table 4:** AMOVA results to test for population structure for *D*. *citri* mtCOI. Values in **bold** are significant with p = 0.00000.

			Among populations			
	Within populations		Among regions		Within regions	
Group Division	% var	F_ST_	% var	F_SC_	% var	F_CT_
Region (n = 5)	14.03	**0.8596**	10.05	**0.1729**	75.92	**0.7592**
N. America/C. America vs.						
The Caribbean						
S. America vs.						
South West Asia vs.						
South East Asia						

### Regional population summary

#### South West Asia population

In all, 413 individuals were successfully sequenced. A total of 16 haplotypes were observed in this region. We observed a higher nucleotide diversity (0.00447), and a lower haplotype diversity (0.218), as compared with the estimated overall nucleotide and haplotype diversity (Table 5). The most common haplotype recovered in this region is haplotype Dcit-6. This haplotype was observed in 364 (88.13%) of SWA individuals. This haplotype was observed in 361/374 (97%) individuals from Pakistan, 3/14 (21.42%) of the individuals from India, and 12/12 (100%) of the psyllids gathered from Colombia. We observed that most of the diversity for the entire dataset belonged within Pakistan and India. Collectively, the haplotype diversity for these two countries was estimated at Hd = 0.149.

**Table 5:** Genetic diversity of *D*. *citri* populations analyzed in this study. Values in **bold** are statistically significant with p = < 0.02. The values on the columns represents Tajima’s D (*D*_*1*_), Fu and Li’s D (*D*_*2*_), Fu and Li’s F (F), sample size (*n*), average number of nucleotide differences (k), segregating sites (S), nucleotide diversity (π), number of haplotypes (h), and haplotype diversity (*H*d).

**Population**	***n***	***D*_*1*_**	***D*_*2*_**	***F***	**k**	**S**	**π**	**h**	***H*d**
1. South America	56	1.60671	0.88016	1.28383	1.17403	3	0.00173	3	0.447
2. South West Asia	413	**-2.27012**	**-5.64237**	**-5.1706**	**0.27800**	**20**	**0.00447**	**16**	**0.218**
3. North and Central America	327	-1.51335	-1.37741	-1.71555	0.41782	9	0.00062	7	0.14
4. The Caribbean	164	-1.12119	0.89984	0.66641	0.65831	4	0.00097	3	0.19
5. South East Asia	148	-0.94481	0.08597	-0.31304	0.59801	6	0.00088	6	0.354

Eight of the sixteen haplotypes were exclusive to Pakistan psyllids (n = 381). Dcit-11 was observed in three individuals from Pakistan and Dcit-16 was observed in two. The remaining five haplotypes (Dcit-9, -10, -14, -16 and -17) seen in samples from Pakistan were recovered from a single individual each. Among the 14 samples from India, six haplotypes were seen in seven psyllids and were found to be exclusive to that country. Haplotype Dcit-23 was observed in two individuals. The remaining five haplotypes (Dcit-24-28) were recovered from a single individual each. All the individuals from Saudi Arabia (n = 8) and Iran (n = 10) showed haplotype Dcit-1. Demographic analyses for this population revealed significant values for Tajima’s D (-2.27; P<0.02), Fu and Li’s D (-5.64: P<0.02), and Fu and Li’s F (-5.17: P<0.02) (Table 5). For SWA populations the Fst value was significant and relatively high (0.86623 P<0.05), suggesting that there is population structure within these populations.

#### South East Asia (SEA) population

In all, 148 sequences were analyzed, and a total of six haplotypes were recovered for psyllids from this region. The haplotype (Hd) and nucleotide (μ) diversity for this region was of 0.354 and 0.00088, respectively. Haplotype Dcit-2 was observed in 118/148 (79.72%) of the SEA individuals, and it was the only haplotype observed in individuals from Taiwan (n = 4), Vietnam (n = 10), Singapore (n = 3) and Mauritius (n = 5). In China, five haplotypes were identified, three of which were exclusive to this country (Dcit-05, Dcit-08, and Dcit-22) and made up a small percent of the sample (4%, 1%, and 1%, respectively). The remaining haplotypes, Dcit-1 and Dcit-2, were observed in eleven individuals (14.67%) and fifty-nine (78.66%) individuals, respectively. Haplotype Dcit-18 was only observed in 9/31 (29.03%) individuals from Thailand. Demographic analyses in this region using haplotype (Hd) and nucleotide (μ) diversity revealed non-significant values for Tajima’s D (-0.94), Fu and Li’s D (0.08), and F (-0.31). The Fst value for this region was significant (P<0.05), and relatively high (0.80376), suggesting the existence of population structure.

#### North and Central America (NCA) population

A total of 327 specimens were successfully sequenced for collections from this region, and seven haplotypes were observed. Six of the haplotypes were recovered from psyllids gathered in a single collection site each. The haplotype and nucleotide diversity of this population was of 0.734 and 0.00294, respectively. The majority of the samples from this region were observed in group B1 311/327 (95.1%). Haplotype Dcit-1 was the most common haplotype in this group; it was seen in 92.66% of the NCA individuals. This haplotype was seen in psyllids from the five geographical regions analyzed. In Edinburg, Texas, U. S. A., 13/119 (10.92%) individuals showed haplotype Dcit-3, consistent with samples gathered in the Caribbean. Haplotype Dcit-4 was only observed in 6 individuals and was unique to a specific location in Yucatan, Mexico. In Los Angeles, California, U. S. A., two individuals showed haplotype Dcit-19. Haplotype Dcit-7, Dcit-20 and Dcit-21 were observed in a single individual each, from psyllids gathered in St. Lucie, Florida, Edinburg, Texas and Los Mochis, Sinaloa, Mexico respectively. Demographic analyses using haplotype (Hd) and nucleotide (μ) diversity estimates resulted in non-significant results for Tajima’s D (-1.51), Fu and Li’s D (-1.37), and F (-1.71). The Fst value for this region was significant (P<0.03), but relatively low (0.10132), suggesting no population structure.

#### South America population (SAM)

Sequences from 56 individuals were included in the analyses. Only three haplotypes were recovered from individuals gathered in this geographic region. A total of 71.43% of the individuals carried haplotype Dcit-2, the most common haplotype found in SEA. This haplotype occurs in 44/56 of the analyzed individuals. These individuals were found in Argentina, Brazil, Paraguay, and Uruguay. Haplotype Dcit-2 is included in group B2. The second most common haplotype, Dcit-6, was found in all individuals from Colombia (12/12) and is common to SWA collections. Haplotype Dcit-6 in included in cluster A. Four individuals, all from a single location in Brazil, carried haplotype Dcit-1. Haplotype Dcit-1 is found in group B1. Demographic analyses in this population using haplotype (Hd) and nucleotide (μ) diversity revealed no significant values for Tajima’s D (1.60), Fu and Li’s D (0.88), and F (1.28). The Fst value for this region was significant (P<0.05) and relatively high (0.81727), suggesting the presence of population structure.

#### Caribbean population

For the Caribbean region, sequences from a total of 164 individuals were analyzed, and three haplotypes were observed. Haplotype Dcit-3 was observed in 89.63% of the collected CAR samples. Additionally, this haplotype was recovered from 13 individuals from Texas. In Puerto Rico, fourteen individuals showed haplotype Dcit-1, which also occurs in NCA, SAM, SWA and SEA. Three individuals (27%) from Guadeloupe exhibited haplotype Dcit-13, which was unique to this region. Demographic analyses in this population using haplotype (Hd) and nucleotide (μ) diversity revealed no significant values for Tajima’s D (-1.12), Fu and Li’s D (0.89), and F (0.66). The Fst value for this region was significant (P<0.002), but relatively low, suggesting no population structure for this geographic region.

## Discussion

### Genetic diversity

A total of 1,108 DNA sequences from the COI mitochondrial region were analyzed, revealing the existence of 28 haplotypes and a haplotype and nucleotide diversity of 0.723 and 0.00294, respectively. Our results are in accordance with previous reports of the relatively low genetic diversity of *D*. *citri* in worldwide populations [27]. Because of this low genetic diversity, these results further suggest that *D*. *citri* is a single species and not a species complex. However, the population structure indicates barriers to gene flow. The numerous private haplotypes observed suggest differentiation and limited gene flow between the five different populations.

It is possible that this genetic isolation is caused by geographic barriers or biotic factors, such as infection with the endosymbiont *Wolbachia*. Recent studies indicated that 66% of all insect species may be infected with this bacterium [44], and *Wolbachia* has been found infecting *D*. *citri*. Infection with *Wolbachia* often causes reproductive abnormalities including cytoplasmic incompability (CI) [45]. It has been suggested that infection with different *Wolbachia* strains may lead to reproductive isolation between populations [46]. An association of COI sequences for *D*. *citri* and *Wolbachia* strains has been observed: U. S. A., Mexico, Belize and American Samoa *D*. *citri* samples were infected with *Wolbachia* strain ST-FL, as well as samples from Pakistan and Colombia [47]. These results suggest that NAM and SWA populations might be infected with the same *Wolbachia* strain. Similarly, *D*. *citri* samples from China, Singapore and Argentina carried *Wolbachia* strain ST-173, a different strain than the one from SWA and NAM. Another strain of *Wolbachia* was associated with samples from the Caribbean, Puerto Rico, Trinidad and Barbados [47]. These results further suggest possible isolation between populations infected with different *Wolbachia* strains.

The information provided in this study is also important for the development of effective biological control programs to control *D*. *citri* populations. The parasitoid *Tamarixia radiata* has been extensively used as a biological control agent for *D*. *citri* [48, 49]. *T*. *radiata* has been successfully introduced to several countries, including Reunion Island, Taiwan, Mauritius, Guadeloupe, Florida, the Philippines and Indonesia, with varied results in parasitism rates of *D*. *citri* [29, 50]. In Reunion, biological control with Pakistani *T*. *radiata* effectively reduced psyllid populations and helped to mitigate the impact of citrus greening [51]. However, when *T*. *radiata* (originating from Taiwan and South Vietnam) was released in Florida, the reported parasitism was lower than 20% [52, 53]. The use of genetic analysis that identify the different evolutionary lineages or strains of *T*. *radiata* and *D*. *citri* [54] may improve parasitism rates and effectiveness of these biocontrol programs. It is possible that *T*. *radiata* populations, as a biological control agent, are more effective for *D*. *citri* management when matched with pests that originate from the same geographic region. However, other ecological factors should be considered to understand variation in rates of parasitism [27].

### Phylogenetic analyses and population structure

Based on the phylogenetic tree and haplotype network it was not possible to determine the ancestral haplotype for *D*. *citri*. Previous analyses in the geographic origin of *D*. *citri* had proposed Dcit-01 as the ancestral haplotype [27, 29], based on frequency and geographic distribution, and suggested a southwestern Asia origin. However, this assumption was based on limited sampling in this geographical region. Previously, only two haplotypes were reported for SWA: Dcit-01 and Dcit-06. In this analysis, additional samples from SWA were included, and a higher diversity was observed, as 13 haplotypes were recovered from this region. Even though Dcit-06 was the most frequent haplotype in SWA it has a limited geographic distribution as it was only observed in Pakistan, and in a few individuals from India and Colombia. The rest of the countries included in SWA (Saudi Arabia n = 8; Iran n = 10) expressed haplotype Dcit-01 only. More sampling from this region, especially from India, where the two haplotypes co-occur, is needed to confirm the geographic origin of *D*. *citri*.

SWA collections exhibited relatively high nucleotide diversity but low haplotype diversity. The haplotype network shows a star-shaped network for this region with a common central haplotype (Dcit-06), and a relatively high number of low-frequency haplotypes. These observations suggest that this is an expanding population, possibly caused by a recent introduction [55]. The diversity estimates generated for each region suggested a possible SEA origin. Relative to SWA (Hd = 0.218), SEA (Hd = 0.354) has high haplotype diversity, low nucleotide diversity, fewer segregation sites, and a high average number of nucleotide differences.

Consistent with results from previous analyses, Dcit-01 was the most geographically distributed haplotype in this study, and was present in all five geographical regions analyzed. However, it was more frequent in NAM populations (92.66%), when compared with the other geographical regions (SWA 7.51%; SAM 7.14%; CAR 8.55%; SEA 10.20%), suggesting a single introduction for NAM. This haplotype was the most frequent haplotype for all the collections from the New World (US, Mexico, Belize, Costa Rica), and it was also present and frequent for some countries in the Old World. In Saudi Arabia and Iran, Dcit-01 was the only haplotype found. It is possible that Dcit-01 originated in SWA, where it is relatively frequent. However, more sampling from this region is necessary to confirm this.

Haplotype Dcit-02 was the most common haplotype for SEA and SAM populations, suggesting that this Asian region is the geographic origin for South America psyllid populations. However, haplotype Dcit-01 was also present in Brazil, where it also co-occurred with Dcit-02, suggesting two independent introductions for this country. It is possible that Dcit-01 was introduced from SWA, or from China or Indonesia, where this haplotype co-occurs with Dcit-02. In addition, haplotype Dcit-06 was found in twelve individuals from Colombia, suggesting a possible third introduction for South America.

Haplotype Dcit-03 was found mostly in the CAR region with thirteen individuals showing this haplotype from Texas collections. Dcit-03 clustered within group B3 along with other haplotypes recovered from Texas (Dcit-20), Guadeloupe (Dcit-13) and India (Dcit-23). Since a closer relative has been found in India (Dcit-23), SWA could be a possible origin for this haplotype. Because this haplotype was found in some of the Texas samples, it is possible that a second introduction from the Caribbean to the U. S. A. occurred.

Even though relatively low genetic diversity was observed, the geographical distribution of the different haplotypes in our study support the existence of (1) five different populations within the different geographic regions: South West Asia (SWA), South East Asia (SEA), North and Central America (NCA), South America (SAM), and the Caribbean (CAR), and (2) four major haplotype groups (Cluster A, subclade group B1group B2 and group B3). A substantial portion of the haplotypes found in this study was unique for each region suggesting limited gene flow among the five geographic regions.
